# Characterising Psycho-Physiological Responses and Relationships during a Military Field Training Exercise

**DOI:** 10.3390/ijerph192214767

**Published:** 2022-11-10

**Authors:** Sean Bulmer, Sean L. Corrigan, Jace R. Drain, Jamie L. Tait, Brad Aisbett, Spencer Roberts, Paul B. Gastin, Luana C. Main

**Affiliations:** 1 Deakin University, Centre for Sport Research, School of Exercise and Nutrition Sciences, Geelong, VIC 3220, Australia; 2 Defence Science and Technology Group, Fisherman’s Bend, Melbourne, VIC 3207, Australia; 3 Deakin University, Institute for Physical Activity and Nutrition (IPAN), School of Exercise and Nutrition Sciences, Geelong, VIC 3220, Australia; 4 La Trobe Sport and Exercise Medicine Research Centre, School of Allied Health, Human Services and Sport, La Trobe University, Bundoora, VIC 3086, Australia

**Keywords:** army, self-report, energy expenditure, MTDS, sleep deprivation, engineers

## Abstract

Over a 15-day period, that included an eight-day field trial, the aims of this study were to (1) quantify the physical workload, sleep and subjective well-being of soldiers in training; (2a) Explore relationships between workload and well-being, and (2b) sleep and well-being; (3) Explore relationships between workload, sleep, and well-being. Methods: Sixty-two Combat Engineer trainees (59 male, 3 female; age: 25.2 ± 7.2 years) wore an ActiGraph GT9X to monitor daily energy expenditure, physical activity, and sleep. Rating of perceived exertion (RPE), sleep quality, and fatigue were measured daily, subjective well-being was reported days 1, 5, 9, 13 and 15. Multi-level models were used for the analysis. Results: Well-being was affected by a combination of variables including workload, subjective sleep quality, sleep duration, and sleep efficiency. RPE and subjective sleep quality were consistently significant parameters within the models of best fit. Conclusions: Perceptions of well-being were lower during the field training when physical workload increased, and sleep decreased. Energy expenditure was comparatively low, while daily sleep duration was consistent with field training literature. Subjective assessments of workload and sleep quality were consistently effective in explaining variations in well-being and represent an efficient approach to monitor training status of personnel.

## 1. Introduction

Military service requires personnel to perform physically and cognitively demanding tasks such as traversing difficult terrain with external load, lifting heavy objects, marksmanship, navigation, tactical assessment, and maintenance of situational awareness in challenging conditions [1,2,3,4,5,6]. In contrast to deployments that may involve volatile, uncertain, complex, and ambiguous environments [7], non-deployed soldiers operate in more controlled and predictable conditions. The challenge of preparing undeployed soldiers for future deployment is overcome, in part, through authentic field-training exercises [8]. Field training for combat arms soldiers, including combat engineers, can include a variety of physical, physiological, and cognitive demands designed to mimic deployment. Further, long hours of sustained operations [8], and sleep deprivation [8,9] are often used to simulate mission requirements, which may include task time constraints and/or night operations. Such experiences cannot be conducted without risk to the individual. Reduced performance capacity, injury or illness, and impaired well-being, can occur during high workload field training [10].

The adverse psycho-physiological effects on personnel during periods of high workload are well documented during periods of military training [8,11]. Field training for 53 h with limited sleep opportunity has been shown to reduce cognitive performance, including vigilance, reaction time, attention, memory, and reasoning [8]. This was associated with a decline in mood states, specifically a decrease in perceptions of vigour, and increased perceptions of fatigue, confusion, depression, and tension [8]. Another study investigating the effects of a three-day sustained operation including road marches, battle drills and restricted sleep found that perceptions of vigour decreased, and tension, depression, confusion, fatigue and anger significantly increased from baseline to the third day [12]. Longer training periods may have potential to exacerbate negative outcomes. Combined, the above factors could impair performance during tasks such as shooting accuracy and reaction time, and may lead to mistakes and injuries due to poor awareness or cognition, potentially rendering personnel ineffective or worse [12,13].

Workload quantification also has a role in managing injury risk in military personnel, as musculoskeletal injuries can reduce the deployable workforce and impair operational capability [14]. For example, the U.S. Army lose ~9% of their deployable force, equivalent to 10 million duty-days each year, through largely preventable injuries [14,15]. Monitoring workloads can provide the opportunity to modify training stress to reduce adverse impacts on well-being and mitigate injury risk [16]. To monitor the effects of workload on athletes, modern training practices use regular recording of perceived training state or well-being, such as the multi-component training distress scale [17]. When such measures have been collected on a serial basis, these have effectively predicted and detected performance capability and training load changes [18,19]. Trends in the responses to each domain in these kinds of measures enable support staff to identify individuals not coping with a workload and make appropriate changes to sustain performance, maintain health, and increase training efficiency [18,19,20]. While there is evidence of workload, sleep, and stress monitoring in military contexts [8,21,22,23], there has been limited work investigating the interrelationships between work, sleep, stress, and fatigue on a serial basis.

A first step toward dedicated well-being and performance management of soldiers is to ascertain whether there are consistent relationships between workloads or sleep, and negative effects such as poor well-being, stress, fatigue, and compromised recovery in cohort and setting. Understanding these relationships could then be leveraged to inform management of workload and recovery strategies to improve soldier well-being and performance. The authors have already demonstrated psychophysiological relationships across 12 weeks of basic military training. Perceived well-being showed relationships most consistently with rating of perceived exertion, and moderate and vigorous physical activity for workload metrics, and perceived sleep quality, sleep duration and sleep efficiency for sleep [24,25]. Whether these relationships hold true in other training environments is yet to be investigated. Therefore, the aim of the present study was to assess whether these same relationships were observed over a 15-day study period, that included four days in the field with at least 24 h of complete sleep deprivation, and four days in a simulated forward operating base. Specifically, (1) Quantify the physical workload, sleep, and subjective well-being of soldiers across an 8-day military training exercise; (2a) Explore relationships between workload and well-being; and (2b) Explore relationships between sleep and well-being; and (3) Explore relationships between workload, sleep, and well-being.

## 2. Materials and Methods

### 2.1. Recruitment and Participants

Data collection occurred as a part of a wider study which also examined hormonal responses [26] and changes in cognitive function [27] over this 15-day period within the Australian Army Combat Engineers Initial Employment Training course at Holsworthy Barracks, NSW, Australia. A conservative power analysis, using Heart Rate Variability (HRV), the ‘noisiest’ variable of interest, was undertaken as part of the wider study revealing a minimum sample size of 19 was required. To account for attrition of up to 30%, a sample size of 25 was sought. Prior to recruitment, the potential risks and benefits of participation were conveyed verbally and in text to potential participants. A written project description was provided by researchers at a group briefing for participants as approved by the Departments of Defence and Veterans’ Affairs Human Research Ethics Committee (protocol number: 021-17). Higher ranking military staff were not present during the briefing, where it was emphasised that participation was voluntary. A total of 64 participants gave written informed consent, and sixty-two completed the data collection with sufficient data to be included in this study (59 male, 3 female; age: 25.2 ± 7.2 years, height: 176.2 ± 10.0 cm, mass: 76.8 ± 15.0 kg). Anthropometric data were collected by researchers immediately following consent. 

### 2.2. Study Design

**Figure 1:**
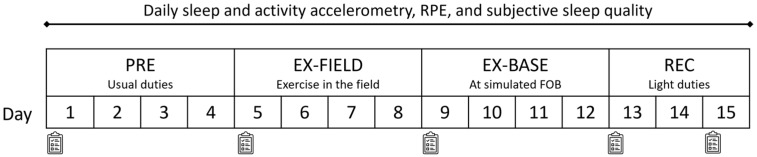
Overview of Study design. Note: clipboard icon indicates completion of MTDS upon awakening.

Data were collected at various points across the 15-day training period, that included eight days in the field (see Figure 1 for study design). On Day 0: soldiers were briefed on device and questionnaire protocols; Days 1–4 (PRE): comprised time in barracks including standard training/activities including bridge building, classroom lessons and teambuilding; Days 5–8 (EX-FIELD): soldiers completed a four-day training phase living in the field performing tasks such as digging trenches, patrolling, simulated mine clearing, and responding to enemy incursions (soldiers were afforded limited sleep opportunity during this time including one night of total sleep deprivation); Days 9–12 (EX-BASE): soldiers completed a four-day training phase at a simulated forward operating base, where they practiced repelling enemy advances and picket duties (sleep was again limited during day and night); Days 13–15 (REC): comprised a three-day in-barracks recovery period with light duties (e.g., cleaning, returning equipment) and habitual sleep opportunities. All daily self-reported measures mentioned below were collected via pen and paper and transferred to electronic copies by researchers. 

### 2.3. Objective Workload

Participants wore an ActiGraph GT9x (ActiGraph, Pensacola, FL, USA) on their non-dominant wrist, with the exception of any periods of water immersion (i.e., showering or swimming) for the duration of the study. ActiGraphs collected 30 Hz data using 60-s epochs. ActiGraphs quantified physical workload through ActiLife proprietary software (ActiLife v6.13; ActiGraph, Pensacola, FL, USA). Objective measures of workload are reported as daily means of energy expenditure (measured in kJ) and percentage of total time spent in zones for sedentary/light (SLPA), and moderate/vigorous (MVPA) physical activity [28]. Daily steps are commonly reported in military literature [29,30] and are used here to measure ambulation across days and compare to previous studies. Non-wear time was determined as more than three h of consecutive ‘0’ total acceleration per day [31]. 

### 2.4. Subjective Workload

Subjective assessment of workload was quantified via Rating of Perceived Exertion (RPE) prior to bed each day [32]. Participants used a category ratio scale (CR-10) of 0 to 10 to rate their exertion for the day where 0 = ‘nothing at all’, 2 = ‘weak’, 5 = ‘strong’, 7 = ‘very strong’ and 10 = ‘very, very strong’ [32]. While this measure has historically been used to rate the intensity of a single training ‘session’, it has been used previously in military settings as a daily measure [30,33]. Responses to all questionnaires in the study were transposed from pen and paper into Microsoft Excel. Data was double entered by two staff. Where discrepancies occurred, data were checked for confirmation of the correct value.

### 2.5. Objective Sleep

The ActiGraph GT9x was used to monitor sleep daily throughout the 15-day monitoring period. Raw data were uploaded via ActiLife software and sleep was assessed using the Cole–Kripke sleep/wake detection algorithm [34], as sleep was opportunistic during field stages of the study period. This algorithm demonstrates 88% agreement (i.e., correctly identified percentages of sleep/wake epochs) compared to polysomnography [34]. Participants recorded prior bed- and get-up times at the end of each day; this was used by researchers to crosscheck sleep/wake times detected via actigraphy [34,35]. Participants often performed night duties during field exercise periods, resulting in bi-phasic overnight sleep. During these periods, sleep duration, wake after sleep onset and awakenings were combined from the end of one main sleep (most often at night) to end of next main sleep across the 24 h window; with sleep efficiency averaged across the sleep opportunities. There was also at least one night of total sleep deprivation. Sleep-related variables of sleep duration (h; total duration of sleep obtained during the 24-h period); sleep efficiency (%; sleep duration expressed as a percentage of time in bed from first attempting to fall asleep and final get-up time); and awakenings (the number of awakenings as determined by Cole et al. note this is a measure of restlessness, recording movements large enough to constitute a likely awakening, but does not indicate consciousness) were recorded for all sleep periods [34]. 

### 2.6. Subjective Sleep Quality

Subjective sleep quality was reported via a 5-point Likert scale (1 “Very good” to 5 “very poor”). Higher scores equate to poorer sleep quality. Lengthier validated measures of sleep quality exist [36]. However, it was key to keep participant contact periods as short as possible, minimising the impost on participants. Therefore, this scale was used for its brevity, with responses provided for the previous 24-h sleep each evening similar to work on wildfire firefighters [37].

### 2.7. Perceived Well-Being

The Multi-component Training Distress Scale is a validated measure of subjective well-being in athlete populations [17,38] and has previously been used in physically demanding workforces (i.e., firefighters; Wolkow et al. [39]). This questionnaire has been found valid and reliable, with internal consistency of the subscales reported as Cronbach alpha ranging from 0.72 to 0.86 [17]. The short 22-item measure was completed via pen and paper upon wakening on days 1, 5, 9, 13, and 15. Items are grouped under six factors: depressed moods, stress, fatigue, vigour, sleep disturbance, and physical symptoms of training (i.e., muscle soreness) were answered on a five-point scale (zero to four: 0 = ‘not at all’, 1 = ‘a little’, 2 = ‘moderately’, 3 = ‘quite a bit’, 4 = ‘extremely’). 

### 2.8. Data Processing and Statistical Analysis

Statistical analyses were completed in the Statistical Product and Service Solutions software (IBM SPSS Statistics for Windows, Version 26.0, 2019, Armonk, NY, USA). Analyses were conducted using a collapsed 4-point data set of mean values to match the phases of the study period (described in ‘study design’ above). ActiGraph 24 h periods (KJ, steps, sleep duration, sleep efficiency and awakenings), and end of day diary entries (RPE, sleep quality) were reduced to means for Days 1–4, Days 5–8, Days 9–12 and Days 13–15 (Referred to as time points PRE, EX-FIELD, EX-BASE, and REC, respectively, hereafter).

For aim one, each variable was assessed using a repeated measures ANOVA, for the 4-point mean values of training phase. Only MTDS Fatigue and MTDS Physical Symptoms of training did not violate sphericity according to Mauchly’s test (*p <* 0.001). To account for violated sphericity while maintaining consistency between reporting of variables, Huynh-Feldt correction was reported in all cases. Post hoc analyses for differences between time-point were conducted via Bonferroni adjustment. 

For aim two (i.e., 2a & 2b) and aim three, Multi-level Linear Models (MLM) assessed potential relationships between the same 4-point mean vales for each training phase. Aim 2a assessed the relationship between well-being and the workload variables, and 2b the relationship between well-being and sleep variables as separate analyses. Aim 3 assessed relationships of MTDS Total Score as the measure of overall well-being, with the combined pool of workload and sleep variables within a single model. MLMs account for potential autocorrelation in the repeated measurements of each participant, as well as serial correlation of data points over time [40]. Variables of workload, and variables of sleep were entered individually into MLM for MTDS Total Score and subscales to identify relationships. Models were then iteratively built adding one variable at a time, to achieve the lowest Akaike’s Information Criterion (AIC) possible, indicating the best goodness of fit. Where a model with less independent variables was similarly effective, the larger model was used, as this includes the model with the greatest amount of data. Models of best fit are presented as β-coefficients with standard deviation (SD), 95% confidence interval (CI), statistical significance within the model, and intercept for effects for each model [41]. 

Some model structures did not include an individual statistically significant effect (*p* < 0.05). However, where the inclusion of a non-significant variable improved the AIC, that variable was deemed to provide a useful mediation of other independent variables, and was therefore retained in the model, unless covariance parameters were violated. Note that both SLPA and MVPA cannot be included in the same model, as the two are opposing portions of 100% physical activity zone time, and including both is essentially including the same variables twice, creating unacceptable covariance within the model.

## 3. Results

### 3.1. Workload 

**Figure 2:**
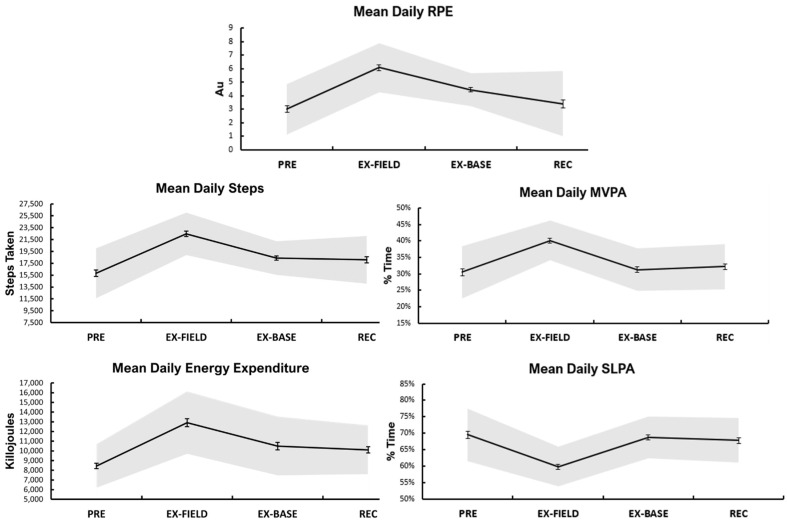
Means per study phase (±SE) for physical workload. NOTE: RPE, rating of perceived exertion; MVPA, moderate to vigorous physical activity; SLPA, sedentary to light vigorous activity; Au, arbitrary units. All variables significantly changed across the four study phases *p* < 0.05.

Participant compliance with available wear time was 94%. Physical workload means (±SE) for the entire training period were: 10,505 (±193) KJ/day, time in SLPA zone 65% (±1%), time in MVPA zone 35% (±1%), 19,095 (±306) steps/day, and a daily RPE of 4.3 (±0.2). A main effect of time was detected for all physical workload variables across the study period (*p* < 0.05, Figure 2). Post hoc analyses revealed KJ/day were higher during EX-FIELD, and lower in PRE than all other phases (all *p* < 0.05). SLPA was lower during EX-FIELD than all other phases (all *p* < 0.05), while MVPA was higher during EX-FIELD than all other phases (all *p* < 0.05). RPE was lower during PRE than EX-FIELD and EX-BASE, and higher in EX-FIELD than all other phases (all *p* < 0.05). Step counts were higher during EX-FIELD than all other phases (all *p* < 0.05). 

### 3.2. Well-Being

**Figure 3:**
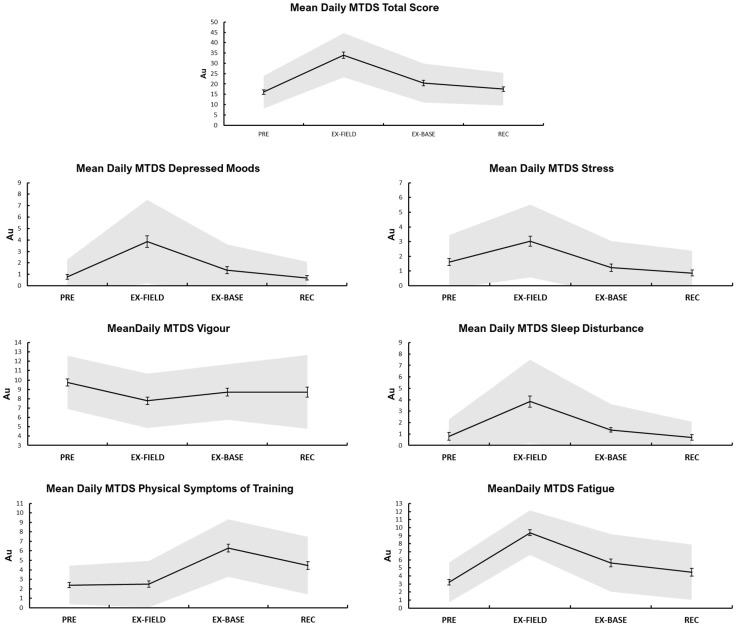
Means per study phase (±SE) for well-being. NOTE: MTDS, Multi-component training distress scale; Au, arbitrary units. All variables significantly affected by time over the four study phases.

Main effects for time were present for MTDS Total Score, as well as all sub-scales (*p* < 0.05) (Figure 3). Post hoc analyses revealed MTDS Total Score was higher during EX-FIELD than all other phases, and lower at PRE and REC than EX-BASE (all *p* < 0.05). MTDS Depressed Moods responses were higher during EX-FIELD than all other phases (all *p* < 0.05). MTDS Stress responses were lower during REC than PRE and EX-FIELD, and higher during EX-FIELD than all other phases (all *p* < 0.05). MTDS Fatigue was lower during PRE than all other phases, higher during EX-FIELD than all other phases, and higher during EX-BASE than REC (all *p* < 0.05). MTDS Vigour was poorer during EX-FIELD than PRE (*p* < 0.05). MTDS Symptoms of Physical Training were lower during PRE than EX-FIELD and EX-BASE, and were higher during EX-FIELD than all other phases (all *p* < 0.05). MTDS Sleep Disturbance was higher during EX-FIELD than all other phases, and lower at REC than PRE and EX-FIELD (all *p* < 0.05).

### 3.3. Sleep 

**Figure 4:**
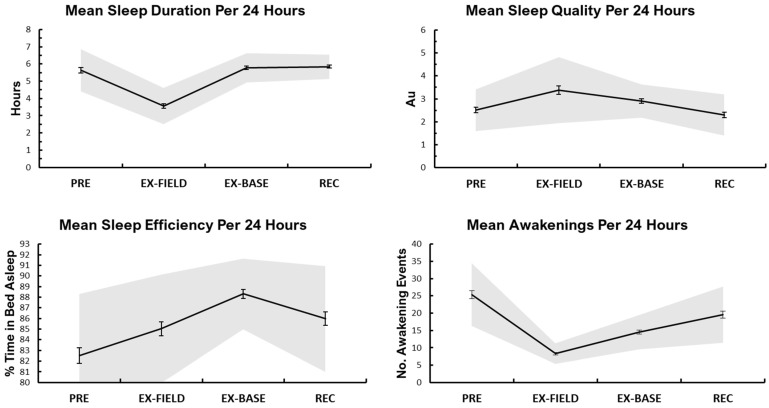
Means per study phase (±SE) for sleep metrics. NOTE: Au, arbitrary units. All variables significantly affected by time across the four study phases *p* < 0.05.

Objective sleep metric means (±SE) during the training period were: 5.4 h (±0.1 h) sleep duration, 84.9% (±0.4%) sleep efficiency, and 19.2 (±0.6) daily awakenings. A main effect of time was detected for sleep efficiency, sleep duration, and awakenings as well as subjective sleep quality across the study period (*p* < 0.05; Figure 4). Post hoc analyses revealed sleep duration was lower during EX-FIELD than all other phases (*p* < 0.05). Sleep efficiency was higher during EX-BASE than all other phases, and higher during REC than PRE and EX-BASE (*p* < 0.05). The number of awakenings were higher during PRE, and lower during EX-FIELD than all other phases; and lower during EX-BASE than REC (all *p* < 0.05). Subjective sleep quality was significantly poorer during EX-FIELD and EX-BASE than PRE and REC (all *p* < 0.05).

### 3.4. Relationships between Workload and Well-Being

Goodness of fit (AIC) was improved in every case by the inclusion of multiple workload variables in each iteration (Table 1). MTDS Total Score and subscales showed the most relationships with RPE as a single measure not including mediation from other variables, and were most often statistically significantly related to RPE within larger models which included this mediation (*p* < 0.05). Only MTDS Sleep and Stress subscales exhibited relationships with a single workload variable (RPE) independent of mediation in a MLM. Physical activity zones effectively moderated the relationship with workload, but only as part of the larger model alongside RPE. Energy expenditure (KJ/day) did not feature as a significant stand-alone relationship, or within a model of best fit once mediated by other variables. The most common final model for MTDS Total Score and subscales relationships with workload parameters was RPE and MVPA (Table 1). 

### 3.5. Relationships between Sleep and Well-Being

Goodness of fit was improved in every case by the inclusion of multiple workload and sleep variables in each iteration (Table 2). MTDS Total Score was significantly related to sleep quality in all three models exhibiting best goodness of fit. RPE, MVPA, sleep quality, and sleep duration were effective contributors to improved goodness of fit in all three models, while sleep efficiency and awakenings could be independently removed without meaningfully reducing goodness of fit. Energy expenditure (KJ/day) did not feature within a model of best fit. 

### 3.6. Relationships between Workload, Sleep and Well-Being

When all workload and sleep variables were considered together, wellbeing was best predicted by a combination of both work and sleep variables (Table 3). Specifically, Timepoint, RPE, MVPA, Sleep Quality, Sleep Duration, Sleep Efficiency and the number of Awakenings.

## 4. Discussion

The first aim of this study was to quantify workload, sleep and well-being during the 15-day study period. Overall, the field training was more challenging for participants than barracks-based training, as evidenced by higher physical workload, alongside poorer sleep, and well-being. The second study aim was to investigate relationships between subjective well-being (MTDS) and workload (Aim 2a), and sleep (Aim 2b). Changes in subjective well-being was best explained by RPE and MVPA as mediating workload factors. The relationship between sleep and well-being were best explained by a combination of measures including subjective sleep quality, sleep duration and sleep efficiency. The third aim was to investigate the relationship between/ the influence of workload and sleep variables on recruit well-being as measured by the MTDS. Variation in MTDS Total Score was best explained by RPE, MVPA, subjective sleep quality, sleep duration, sleep efficiency, and awakening. Energy expenditure was not included in any model of best fit. Subjective sleep quality was the most common effective single measure and model contributor, followed by sleep duration. 

### 4.1. Physical Workload 

EX-FIELD demonstrated the highest energy expenditure, RPE, MVPA and step counts when compared to all other periods (Figure 1). This is consistent with previous studies of military field training which reported high energy expenditure, RPE, MVPA and steps for soldiers when compared to barracks-based training [30,42]. In the present study, the higher MVPA observed during EX-FIELD is likely attributable to the combat scenarios which involved repeated enemy contact. Many tasks performed during EX-FIELD required prolonged attentiveness (guard/picket, mine clearing), and sleep opportunity was minimal during this phase. This may explain the higher steps observed during EX-FIELD due to these longer waking periods and sustained activity when compared to other phases. 

The energy expenditure during EX-FIELD in the present study (i.e., 12,905 ± 411 kJ/day) was lower than what was reported in Finnish soldiers over a seven-day period that included four days of field training (i.e.,16,600 kJ/day) [43]. However, it was similar to the 13,200 kJ/day reported in U.S. Army recruits over the final 20 day period of basic training which included seven days of field training [44]. The differences in energy expenditure across studies highlights a need to either track training cohorts directly, or at least gather data on the specific cohort and training exercise if precise energy expenditure is requred for workload, recovery or nutirition management during field training periods. A generalised approach or estimation is unlikely to be effective if precision is important. 

Steps per day of 19,095 (±306) in this study are higher than the 13,937 (±2276) reported in a Finnish 21-day field exercise [30] as well as the 13,459 (±4376) steps per day across the 10-week U.S. Army Basic Combat Training [44]. The site of the accelerometer may explain, at least in part, the observed differences as Ojanen et al. [30] and Alemany et al. [44] attached the acceleromter to the hip, as opposed to the wrist in the current study. The observed difference between studies could potentially also be due to longer periods of wakefulness and patrolling in the current study; however noting the lower energy expenditure in comparison to the other studies, this suggests that most steps were taken at a low physical activity intensity. This supports the premise that although step counts can be useful in identifying ambulation or distance covered, step-count as a discrete measure to identify levels of physical activity or daily effort in military settings, should not be relied upon as a single physical activity indicator. 

### 4.2. Sleep

Mean sleep duration was 5.4 h (±0.1 h) across the study period, however 3.6 h (±0.1) was observed during EX-FIELD. This is consistent with 3 h (±0.3) sleep per night during U.S. Army recruit field training [8]. Sleep duration this low over an extended period can be common in military cohorts, especially during field periods [45,46,47]. Sleep opportunity was intentionally restricted during EX-FIELD with one night of no sleep opportunity due to night operations. However, any sleep duration under 7 h in adults will likely begin to accrue a sleep debt over subsequent nights of reduced sleep, with the potential to disturb mood (as noted in ‘well-being’ below), and degrade physical and cognitive performance [48]. This could adversely affect a soldier’s ability to perform at a high level [8]. However, exposure to restricted sleep during military training is often deemed vital to simulate potential operational conditions, such as a reduction of sleep during British officer cadet training from 5.6 h (±1.5) daily on base, to 2.1 h (±1.3) during field work [46]. 

Subjective sleep quality was also significantly poorer during EX-FIELD and EX-BASE, potentially due to the nature of field training, such as night operations, uncomfortable sleeping conditions and other environmental factors, which differ to barracks sleep. Sleep efficiency was lowest in PRE, and highest during EX-BASE, which is likely the consequence of increase sleep pressure following the accrued sleep debt during the preceding period (EX-FIELD). Good sleep efficiency is commonly recognised as 85% or higher [49], which was not achieved in this study period. Regardless, sleep efficiency never fell below 80%, which is surprising considering the nature of the sleeping environment.

### 4.3. Well-Being

All MTDS subscales and the Total Score changed significantly across the study period, and the increases in MTDS Stress, Fatigue and Depressed Moods (Figure 1) during EX-FIELD are consistent with the exiting literature. Field training has a demonstrated effect of mood disturbance and fatigue [8], and reductions in physical capability [30]. However, in the current study all subscales were negatively affected by EX-FIELD except ‘physical symptoms of training’ which did not increase until EX-BASE. Measures of muscle soreness (similar to MTDS Physical Symptoms of Training) have been shown to increase when load and EE increase during strenuous winter military training [50] and with higher total training volume [51]. However, delayed onset muscle soreness can take several days to manifest [52], which may explain the rise during EX-BASE compared to EX-FIELD. The current results demonstrate that the MTDS total and subscales are sensitive to changes in workload and sleep during military field training, indicating potential utility as a monitoring tool.

### 4.4. Relationships between Physical Workload and Well-Being

In the case of MTDS Total Score and subscales in this study, a combination of RPE and MVPA achieved the best goodness of fit between well-being and workload (Table 1). Energy expenditure, while extremely valuable for contextualising activity across days, was not meaningfully related to changes in well-being through MLM analysis. RPE was the only workload variable to effectively relate to well-being (MTDS subscales of Stress and Sleep Disturbance) without mediation from another variable (Table 1). It was also the most commonly statistically significant variable within MLMs. Therefore, RPE was the best stand-alone measure of perceived workload related to well-being in soldiers, which is consistent with previous evidence regarding the sensitivity of RPE to changes in training load [18,53]. 

One of the limitations of using actigraphy in military contexts is that it does not capture the additional weight carried by personnel (e.g., equipment, pack, etc.), the difficult terrain they may be traversing, or weight of objects moved or used. Each of these factors independently and collectively can add physiological demand to movement [54]. Therefore, reliance on actigraphy may create an underestimation of physical workload. However, daily RPE may be able to infer the extra physical effort elicited when carry external loads or traversing difficult terrain. This may explain why the combination of RPE and MVPA increases goodness of fit with the measures of well-being. The result of this analysis advocates for the use of a combination of subjective (RPE) and objective (MVPA) measures of workload, to understand the accumulative strain on an individual. However, where objective measurement (i.e., actigraphy) is not feasible, subjective strain perception of effort (RPE) as the measure of workload had the best relationship with well-being in this study. These relationships with workload support the sensitivity and potential utility of the MTDS as a cost and time-effective measure in monitoring of soldiers in field training. This could be extended to operational environments with further investigation.

### 4.5. Relationships between Sleep and Well-Being

MTDS domains, as a reflection of various facets of well-being, were most commonly related to subjective sleep quality and sleep duration (Table 2). Sleep is a key requirement for individuals to recover from demanding workloads [55], and the quality and duration of a sleep period having greatest influence on well-being during the study period is consistent with this. In every model, non-significant contributions from sleep variables still improved the overall goodness of fit, reinforcing the moderating effect of sleep on well-being. The MTDS Total Score and MTDS Stress subscale were best explained by the full suite of sleep metrics in a single model, while the MTDS Fatigue, Depressed Moods, Vigour and Sleep subscales were best explained by models comprising subjective sleep quality, sleep duration and either sleep efficiency or awakenings. While each sleep variable improved goodness of fit in models with at least one MTDS subscale, goodness of fit was only improved by the inclusion of ‘all’ sleep measures in association with MTDS Stress. Links to sleep here are likely in part due to perception of stress’ know relationship with increases in sympathetic nervous system activity, which alters hormone balance, increasing arousal [56]. Poor sleep alongside perception of stress during military training periods has been established previously [8], and again appears to be the MTDS domain most susceptible to changes in sleep parameters. Further investigation as the potential for, and specifics of a bi-directional stress/sleep relationship in this cohort would be beneficial to stress and sleep monitoring.

Subjective sleep quality was the most common variable present in models of best fit and was significantly related to MTDS Total Score as a single variable. However, no sleep metric was set apart as the single best moderator of well-being across all domains in these analyses. The combination of subjective sleep quality and objective sleep metrics provided by actigraphy appears best if aiming to relate sleep to multiple domains of well-being in the military field training setting. This could be due to the various stressors, sleep disruptions and sleep environments in the field setting, which alter sleep in multiple ways that a single sleep metric will not detect. This finding supports the use of actigraphy as a solution to provide a variety of sleep metrics in a relatively unintrusive manner [9,12]. However, if objective measures are not feasible, the findings of the current study support the use of subjective sleep quality as an effective single measure to monitor the potential impact of training on well-being.

### 4.6. Relationships between Combined Workload and Sleep Variables, and Well-Being

Consistent with the models of workload and sleep as separate concepts, the models of best fit for MTDS Total Score comprised all workload and sleep variables apart from energy expenditure. It is important to recognise that without further investigation in similar settings, it cannot be discounted that these variables have important interactions within models under specific circumstances, and so their potential utility in relating to well-being should be noted. A key difference between the combined workload and sleep model and earlier models, is a lack of significance (*p* > 0.05) of RPE and MVPA within the combined model. This indicates that workload’s interrelationships with sleep variables were not able to greatly increase the strength of relationships in this case. On its own, subjective sleep quality appears the most consistently effective single measure when relating to well-being scores both as a contributor within some of the larger models, and as a stand-alone measure. With further refinement, such as the identification of indicative thresholds, subjective sleep quality may provide a low-burden option to monitor soldiers during field training.

One inherent limitation to note is the inability of actigraphs and the algorithms used to detect any additional energy expenditure as a result of additional weight carried, and the terrain traversed. Similarly, acknowledging that 100% wear time cannot be guaranteed it is possible that energy expenditure may be less that what was actually recorded. However, in the current study, 70% of available daily wear time was required for inclusion in the analysis [57], which is consistent with recommendations to most accurately reflect daily activity. A second limitation is the potential for participants to under-report their perceptions of wellbeing, exertion, and sleep quality. However, participants were made aware that no Defence personnel would see individual results, which anecdotally allayed concerns and likely yielded accurate responses.

## 5. Conclusions

Physical workload, sleep and well-being were quantified across the 15-day period. Workload increased and perceived well-being was negatively affected during military field training compared to in-barracks training. Mean energy expenditure across the study period was lower than previously reported in military studies, but an average sleep duration of 5.4 h per night over the 15-day study period was consistent with previous results. This sleep duration is likely to impair soldiers physical and cognitive capacity. However, this is a realistic demand of deployed operations, and is necessary exposure to prepare for deployments. Relationships between well-being workload and sleep were assessed, revealing that perceived well-being, as measured by MTDS, were sensitive to changes in physical workload and sleep across the training period, and were best explained by combinations of subjective (RPE and subjective sleep quality) and objective (MVPA, sleep duration, sleep efficiency and awakenings) workload and sleep variables. If objective monitoring measures are not feasible, the most effective single measures appear to be RPE to infer current workload effects on well-being, and subjective sleep quality to infer overall well-being related to sleep.

## Figures and Tables

**Table 1:** Parameters of multi-level linear models of best fit, and significant single measure relationships for energy expenditure, MVPA and RPE with MTDS.

	Parameter	AIC	*β* Estimate	95% CI Lower	95% CI Upper	*p*
**MTDS Total Score**						
Model of best fit	Intercept	1392	17.26	8.82	25.71	**<0.001**
	Timepoint		3.77	2.35	5.18	**<0.001**
	RPE		0.503	−0.33	1.33	0.237
	MVPA		−19.56	−42.54	3.41	0.095
**MTDS Fatigue**						
Model of best fit	Intercept	961	2.84	0.30	5.37	**0.028**
	Timepoint		1.61	1.19	2.02	**<0.001**
	RPE		0.06	−0.18	0.31	0.625
	MVPA		−5.29	−12.20	1.61	0.132
**MTDS Depressed Moods**						
Model of best fit	Intercept	900	2.83	0.69	4.96	**0.010**
	Timepoint		0.49	0.14	0.83	**0.006**
	RPE		0.15	−0.06	0.36	0.161
	MVPA		−8.30	−14.16	−2.45	**0.006**
**MTDS Vigour**						
Model of best fit	Intercept	918	7.25	5.01	9.50	**<0.001**
	Timepoint		0.35	−0.01	0.72	0.060
	RPE		−0.05	−0.27	0.16	0.626
	MVPA		−1.99	−8.12	4.14	0.522
**MTDS Physical Symptoms of Training**						
Model of best fit	Intercept	896	1.76	−0.34	3.87	0.101
	Timepoint		1.13	0.79	1.47	**<0.001**
	RPE		−0.01	−0.22	0.19	0.872
	MVPA		−1.60	−7.40	4.18	0.585
**MTDS Sleep**						
Effective single measure	Intercept	938	1.15	−0.00	2.31	0.051
	Timepoint		−0.04	−0.32	0.25	0.801
	RPE		0.17	0.00	0.34	**0.045**
Model of best fit	Intercept	883	1.38	−0.64	3.41	0.180
	Timepoint		−0.05	−0.37	0.27	0.744
	RPE		0.15	−0.04	0.35	0.127
	MVPA		−0.26	−5.84	5.32	0.926
**MTDS Stress**						
Effective single measure	Intercept	847	0.54	−0.37	1.45	0.244
	Timepoint		0.21	−0.02	0.45	0.084
	RPE		0.17	0.04	0.31	**0.010**
Model of best fit	Intercept	791	1.78	0.21	3.36	**0.026**
	Timepoint		0.23	−0.03	0.49	0.087
	RPE		0.22	0.07	0.38	**0.004**
	MVPA		−4.38	−8.67	−0.10	**0.045**

Note: bolding highlights = *p* < 0.05; *β* = beta estimate; *p* = significance value; 95% CI = *β* 95% Confidence Interval.

**Table 2:** Parameters of multi-level linear models of best fit, and significant single measure relationships for sleep quality, sleep duration, sleep efficiency and awakenings with MTDS.

	Parameter	AIC	*β* Estimate	95% CI Lower	95% CI Upper	*p*
**MTDS Total Score**						
Effective measure	Intercept	1668	7.56	2.32	12.81	**0.005**
	Timepoint		3.65	2.47	4.83	**<0.001**
	Sleep Quality		1.73	0.42	3.03	**0.010**
Model of best fit	Intercept	1525	12.49	−26.06	51.04	0.524
	Timepoint		3.30	1.92	4.69	**<0.001**
	Sleep Quality		2.00	0.58	3.43	**0.006**
	Sleep Duration		2.41	0.58	4.39	**0.011**
	Sleep Efficiency		−0.16	−0.63	0.31	0.513
	Awakenings		−0.24	−0.54	0.07	0.132
**MTDS Fatigue**						
Effective measure 1	Intercept	1065	−0.28	−2.10	1.54	0.761
	Timepoint		1.56	1.17	1.96	**<0.001**
	Sleep Duration		0.33	0.01	0.65	**0.041**
Effective measure 2	Intercept	1062	−8.02	−15.66	−0.38	**0.040**
	Timepoint		1.49	1.08	1.90	**<0.001**
	Sleep Efficiency		0.11	0.02	0.21	**0.017**
Model of best fit 1	Intercept	1054	−9.47	−17.19	−1.76	**0.016**
	Timepoint		1.48	1.07	1.88	**0.000**
	Sleep Quality		0.43	−0.00	0.86	0.051
	Sleep Duration		0.40	0.056	0.75	**0.023**
	Sleep Efficiency		0.09	−0.00	0.19	0.056
Model of best fit 2	Intercept	1054	−2.17	−4.76	0.42	0.100
	Timepoint		1.44	1.02	1.85	**<0.001**
	Sleep Quality		0.42	−0.01	0.85	0.055
	Sleep Duration		0.74	0.32	1.15	**0.001**
	Awakenings		−0.07	−0.13	−0.00	**0.036**
**MTDS Depressed Moods**						
Effective measure	Intercept	988	−0.53	−2.01	0.95	0.482
	Timepoint		0.40	0.08	0.72	**0.014**
	Sleep Duration		0.26	0.00	0.52	**0.046**
Model of best fit 1	Intercept	981	−3.39	−9.78	3.00	0.296
	Timepoint		0.39	0.06	0.73	**0.021**
	Sleep Quality		0.39	0.04	0.75	**0.030**
	Sleep Duration		0.36	0.07	0.64	**0.014**
	Sleep Efficiency		0.01	−0.06	0.09	0.705
Model of best fit 2	Intercept	982	−2.16	−4.31	−0.02	**0.048**
	Timepoint		0.37	0.03	0.70	**0.035**
	Sleep Quality		0.39	0.03	0.74	**0.034**
	Sleep Duration		0.45	0.11	0.79	**0.010**
	Awakenings		−0.02	−0.07	0.03	0.434
**MTDS Vigour**						
Model of best fit 1	Intercept	1011	4.53	−2.39	11.45	0.199
	Timepoint		0.28	−0.09	0.64	0.136
	Sleep Quality		0.19	−0.19	0.58	0.329
	Sleep Duration		0.18	−0.13	0.49	0.252
	Sleep Efficiency		0.00	−0.08	0.09	0.917
Model of best fit 2	Intercept	1012	4.86	2.52	7.19	**<0.001**
	Timepoint		0.30	−0.08	0.67	0.118
	Sleep Quality		0.19	−0.19	0.58	0.320
	Sleep Duration		0.16	−0.21	0.54	0.390
	Awakenings		0.01	−0.05	0.06	0.837
**MTDS Physical Symptoms of Training**						
Model of best fit	Intercept	992	−0.21	−1.72	1.30	0.782
	Timepoint		1.01	0.69	1.34	**<0.001**
	Sleep Duration		0.32	0.06	0.59	**0.017**
Model of best fit	Intercept	985	3.47	−3.01	9.95	0.292
	Timepoint		1.12	0.77	1.46	**<0.001**
	Sleep Quality		0.31	−0.05	0.67	0.089
	Sleep Duration		0.46	0.16	0.75	**0.002**
	Sleep Efficiency		−0.06	−0.14	0.01	0.109
**MTDS Sleep**						
Effective measure 1	Intercept	1038	0.29	−0.93	1.52	0.637
	Timepoint		0.07	−0.20	0.33	0.628
	Sleep Quality		0.46	0.16	0.77	**0.003**
Effective measure 2	Intercept	971	−4.50	−10.53	1.53	0.143
	Timepoint		−0.12	−0.43	0.20	0.460
	Sleep Efficiency		0.08	0.00	0.15	**0.040**
Effective measure 3	Intercept	968	2.88	1.76	4.04	**<0.001**
	Timepoint		−0.08	−0.38	0.21	0.589
	Awakenings		−0.06	−0.09	−0.02	**0.004**
Model of best fit 1	Intercept	962	−5.86	−11.91	0.20	0.058
	Timepoint		−0.06	−0.38	0.25	0.697
	Sleep Quality		0.41	0.07	0.74	**0.018**
	Sleep Duration		−0.12	−0.38	0.15	0.390
	Sleep Efficiency		0.08	0.01	0.16	**0.025**
Model of best fit 2	Intercept	962	0.92	−1.11	2.95	0.374
	Timepoint		−0.10	−0.42	0.22	0.543
	Sleep Quality		0.39	0.06	0.73	**0.023**
	Sleep Duration		0.19	−0.13	0.51	0.238
	Awakenings		−0.06	−0.11	−0.01	**0.013**
**MTDS Stress**						
Effective measure	Intercept	931	0.19	−0.77	1.15	0.696
	Timepoint		0.25	0.03	0.46	**0.023**
	Sleep Quality		0.34	0.10	0.58	**0.005**
Model of best fit	Intercept	869	7.88	0.71	15.04	**0.031**
	Timepoint		0.24	−0.01	0.50	0.062
	Sleep Quality		0.32	0.05	0.58	**0.019**
	Sleep Duration		0.41	0.06	0.76	**0.023**
	Sleep Efficiency		−0.10	−0.19	−0.01	**0.028**
	Awakenings		−0.07	−0.13	−0.01	**0.014**

Note: bolding highlights = *p* < 0.05; *β* = beta estimate; *p* = significance value; 95% CI = *β* 95% Confidence Interval.

**Table 3:** Parameters of multi-level linear model of best fit for Multi-Component Training Distress Scale Total Score with combined workload and sleep variables.

	Parameter	AIC	*β* Estimate	95% CI Lower	95% CI Upper	*p*
Model of best fit	Intercept	1354	6.05	−39.62	51.71	0.794
	Timepoint		3.34	1.79	4.90	**<0.001**
	RPE		0.47	−0.47	1.40	0.327
	MVPA		−15.14	−41.09	10.82	0.251
	Sleep Quality		2.12	0.55	3.68	**0.008**
	Sleep Duration		1.96	−0.25	4.16	0.082
	Sleep Efficiency		−0.04	−0.57	0.50	0.898
	Awakenings		−0.13	−0.53	0.26	0.500

Note: bolding highlights = *p* < 0.05; *β* = beta estimate; *p* = significance value; 95% CI = *β* 95% Confidence Interval.

## Data Availability

Data from this study is not publicly available however access may be requested from DST.
